# Data sets of predicted stable and meta-stable crystalline phase structural of NB-N system under pressure

**DOI:** 10.1016/j.dib.2020.106054

**Published:** 2020-07-22

**Authors:** Diego Restrepo-Leal, José Sierra-Ortega, Gene Elizabeth Escorcia-Salas

**Affiliations:** Grupo de Investigación en Teoría de la Materia Condensada, Universidad del Magdalena, Facultad de Ciencias Básicas, Carrera 32 No 22 – 08, Santa Marta, Colombia

**Keywords:** Convex hull, Crystal structure, Meta-stable phases, Prediction, Stable phases

## Abstract

This document presents a dataset on various stoichiometric Niobium nitrides compounds under different pressures, which have been identified by first-principles calculations in combination with an evolutionary algorithm methodology implemented in the USPEX code in its variable-composition mode. The feature of this methodology is to find the ground state or metastable structures with only the knowledge of chemical composition at given pressure conditions and predict through all possible structures, not relying on any prior known structural information. We have successfully predicted the crystal structures and phase transitions of *NbN* at pressures up to 100 GPa. Because the Niobium nitrides represent a rich family of phases where the stability and microstructures are still not completely understood, it is exciting to find news structures of *Nb_x_N_y_* under high pressure.

**Specifications Table**

**Table:** 

**Subject**	Materials Science.
**Specific subject area**	Materials Science (General), Metals and Alloys, Computer Science Applications.
**Type of data**	Table, Figures.
**How data were acquired**	The stable crystal structures and compositions of *Nb_x_N_y_* compounds at 0, 30, 50, and 100 GPa were predicted by using the evolutionary algorithm USPEX (Universal Structure Predictor: Evolutionary Xtallography) software together with the Quantum-ESPRESSO package. Stable compositions were determined using the convex hull construction; taking into account that a compound is thermodynamically stable when its enthalpy of formation from the elements and from any other compounds is negative. Formation enthalpy calculations and structure relaxations were performed by using density functional theory (DFT) within the Perdew-Burke-Ernzerhof (PBE) generalized gradient approximation (GGA) [1], as implemented in the Quantum-ESPRESSO code [2].
**Data format**	Raw, filtered and analyzed.
**Parameters for data collection**	To generate the structures, we used typical parameters for USPEX calculations, with which efficiency is known to be very high. The USPEX input file was configured in its variable composition mode to produce *Nb_x_N_y_* structures with a maximum of 30 atoms in the primitive cell. These conditions were replicated for each pressure conditions. In each case, two relaxation processes were implemented with Quantum-ESPRESSO, the first one with a precision of 4 × 10^−6^ and the second one with a precision of 1 × 10^−6^.
**Description of data collection**	During the structure searching, the first generation contained 20 candidate structures is produced randomly, considering that all possible stoichiometries in the bulk were allowed for up to 30 atoms in the primitive cell. In the succeeding generations, each generation contained 20 structures, which were obtained by applying 20% heredity, 20% randomly, 20% softmutacion, 20% transmutation respectively.
**Data source location**	Universidad del Magdalena, Santa Marta, Magdalena, Colombia.
**Data accessibility**	Repository name: Complementary material: Data sets of predicted stable and meta-stable crystalline phase structural of *Nb*-*N* system under pressure Data identification number: 10.17632/26b8njvyc8.3 Direct URL to data: https://data.mendeley.com/datasets/26b8njvyc8/3

**Value of the data**

• The phases obtained are important to better understand the physical essence of *Nb_x_N_y_* and its practical engineering applications. Knowing the structure, a large number of properties of a material can be calculated, even before it is synthesized, hence the usefulness or crucial importance of the data present here.
• Niobium nitride have been shown to possess interesting properties [3,5], but these researches have always been conducted under ambient pressure. Whereby the data of different crystal structures of *Nb_x_N_y_* under environmental conditions and high pressures will lay a foundation for both theoretical and experimental researchers for characterization analysis and further technology evaluation of this compound.
• These data shown that the *Nb_x_N_y_* can possess multiple stoichiometries and structures under different pressures. Understanding the structure of materials is crucial for understanding their properties and potential applications. Researchers can make use of the data presented to characterize these structures.
• Determination of the crystal structure of most materials at normal conditions could be trivial by experimental techniques. However, at extreme conditions, the same treatment becomes extremely problematic, and computer simulation becomes essential for obtaining structural information. Not only at extreme but also at normal conditions crystal structure prediction is of enormous value and a necessary key step in computational materials discovery.

## 1 Data description

The data here reported correspond to 532 phases of the *Nb*-*N* system at different pressures (135 at 0 GPa, 130 at 30 GPa, 134 at 50 GPa and 135 at 100 GPa), which were performed using evolutionary algorithm methodology implemented in the USPEX [6,8] code in its variable composition mode [9,10]. All these data are included in the files: Individuals_0 GPa, Individuals_30 GPa, Individuals_50 GPa, and Individuals_100 GPa of the complementary material.

Some structures mentioned above make up a convex hull for each pressure, which is defined as the Gibbs free energy of formation of the most stable phases at each composition, as seen in equation 1:

(1) $$Y=\Delta G\left(Nb_{x}N_{y}\right)=\frac{\left[G\left(Nb_{x}N_{y}\right)-xG\left(Nb\right)-yG\left(N\right)\right]}{\left(x+y\right)}$$

Where, $Y=\Delta G(Nb_{x}N_{y})$ is the formation enthalpy of the *Nb_x_N_y_* system, *x* and *y* are the number of *Nb* and *N* atoms respectively.

Thus, of the 135 structures generated for the pressure 0 GPa, 70 of them are present in the convex hull displayed in the following figure.

The characteristics of the first 10 phases include in the convex hull at 0 GPa are listed in Table 1.

**Fig. 1:**
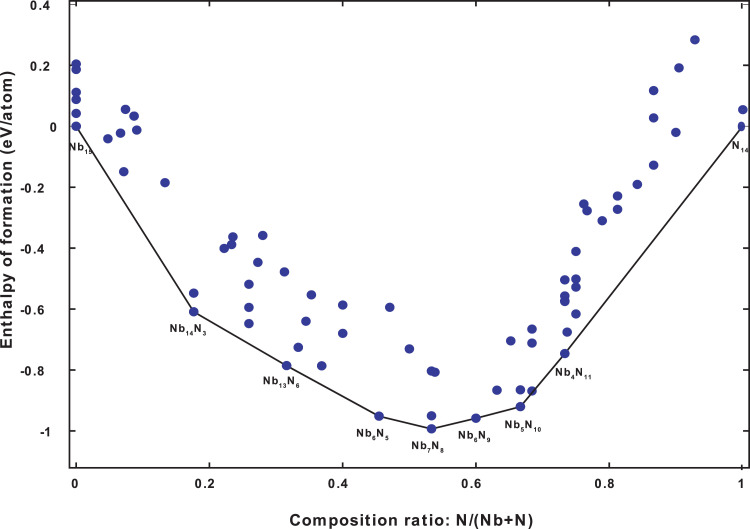
The Convex hull diagram of the *Nb-N* system at 0 GPa.

**Table 1:** Formation enthalpy Y (eV/atom), Composition ratio X, Symmetry S, Fitness F (eV/block), Volumes V(Å^3^/atom), Enthalpies E(eV/atom), and Compositions C for the first 10 structures include in the convex hull of the Nb-N system at 0 GPa.

**ID**	**C**	**E**	**V**	**F**	**S**	**X**	**Y**
71	[7,8]	−893.5888	14.4958	0.0000	1	0.533	−0.9929
75	[0 14]	−270.1198	14.3751	0.0000	1	1.000	0.0000
89	[14 3]	−1369.2158	18.0875	0.0000	1	0.176	−0.6086
97	[15 0]	−1603.9973	22.8193	0.0000	1	0.000	0.000
113	[13 6]	−1183.5582	16.1519	0.0000	1	0.316	−0.7854
121	[6,9]	−804.6286	14.1712	0.0000	1	0.600	−0.9578
123	[5,10]	−715.6656	13.0397	0.0000	1	0.667	−0.9200
125	[4,11]	−626.5662	18.2131	0.0000	1	0.733	−0.7457
132	[6,5]	−998.6407	14.8818	0.0000	1	0.455	−0.9513
66	[6 13]	−692.2129	10.5534	0.0055	1	0.684	−0.8686

**Fig. 2:**
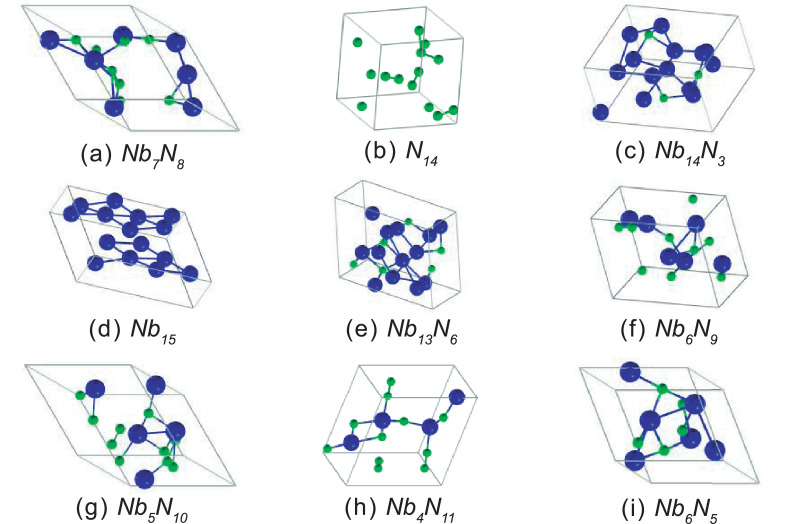
Crystal structures of first nine phases present in the convex hull at 0 GPa. The blue balls are Nb atoms and the green balls are N atoms.

The Crystal structures of the firsts nine phases presented in Table 1 are schematically shown in Fig. 2.

**Fig. 3:**
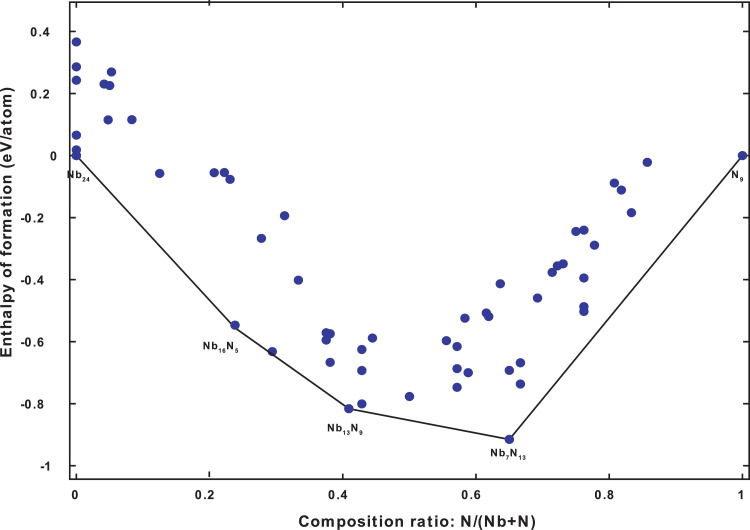
The Convex hull diagram of the Nb-N system at 30 GPa.

For the Case 30 GPa, of the 130 structures generated, 59 of them are present in the convex hull shown in Fig. 3.

**Fig. 4:**
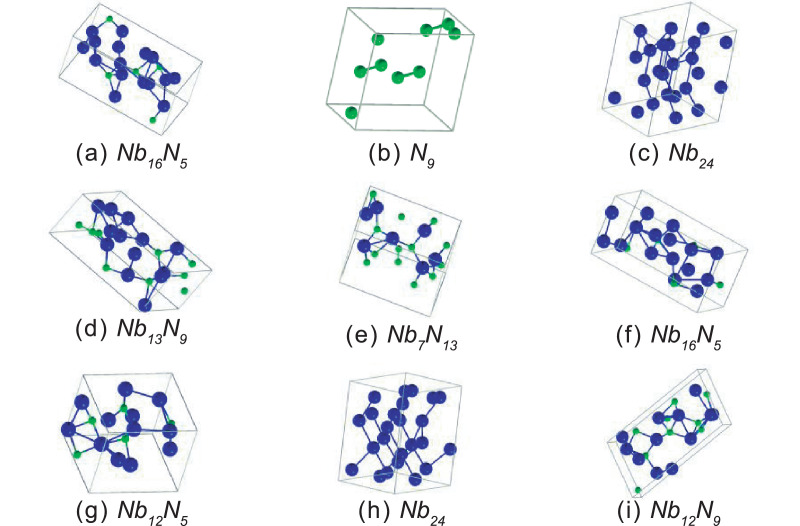
Crystal structures of first nine phases present in the convex hull at 30 GPa. The blue balls are Nb atoms and the green balls are N atoms.

In the Table 2 are listed the characteristics of the first 10 structures presented in the convex hull at 30 GPa.

**Table 2:** Formation enthalpy Y (eV/atom), Composition ratio X, Symmetry S, Fitness F (eV/block), Volumes V(Å^3^/atom), Enthalpies E(eV/atom), and Compositions C for the first 10 structures include in the convex hull of the Nb-N system at 30 GPa.

**ID**	**C**	**E**	**V**	**F**	**S**	**X**	**Y**
32	[16 5]	−1287.1755	18.1217	0.0000	1	0.238	−0.5553
75	[0 9]	−270.1825	16.1194	0.0000	1	1.000	0.0000
105	[24 0]	−1604.2570	23.0125	0.0000	1	0.000	0.0000
107	[13 9]	−1059.3153	14.4932	0.0000	1	0.409	−0.8161
116	[7 13]	−738.0236	16.5452	0.0000	1	0.650	−0.9150
112	[16 5]	−1287.1669	18.1217	0.0086	1	0.238	−0.5467
95	[12,5]	−1212.5120	16.4024	0.0089	1	0.294	−0.6318
81	[24 0]	−1604.2386	23.0125	0.0184	1	0.000	0.0184
85	[12,9]	−1033.3115	16.8773	0.0234	1	0.429	−0.8007
51	[24 0]	−1604.1912	23.0125	0.0658	1	0.000	0.0658

The following figure shows the crystal structures of the firsts nine phases presented in Table 2.

**Fig. 5:**
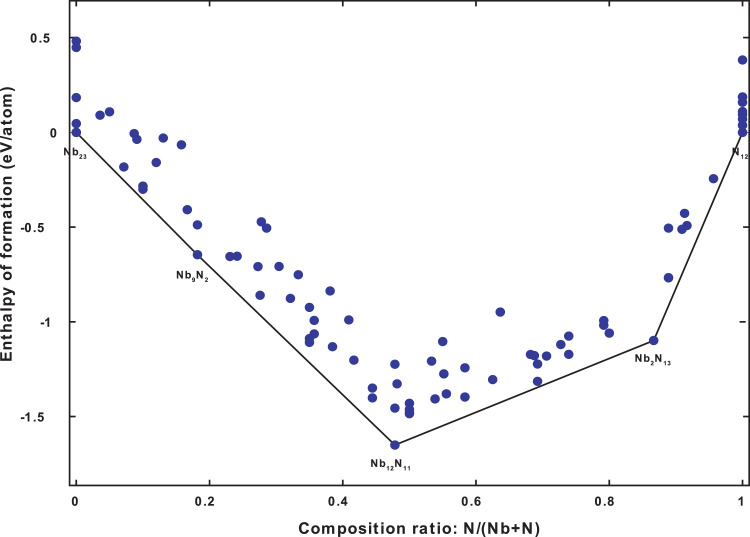
The Convex hull diagram of the Nb-N system at 50 GPa.

For 50 GPa, of the 134 structures generated, 82 of them are present in the convex hull shown in Fig. 5.

The characteristics of the first 10 structures presented in the convex hull at 50 GPa are listed in Table 3.

**Table 3:** Formation enthalpy Y (eV/atom), Composition ratio X, Symmetry S, Fitness F (eV/block), Volumes V(Å^3^/atom), Enthalpies E(eV/atom), and Compositions C for the first 10 structures include in the convex hull of the Nb-N system at 50 GPa.

**ID**	**C**	**E**	**V**	**F**	**S**	**X**	**Y**
8	[23 0]	−1604.0754	22.8215	0.0000	2	0.000	0.000
18	[9,2]	−1362.0233	19.9797	0.0000	8	0.182	−0.6457
69	[2 13]	−448.3141	15.0906	0.0000	1	0.867	−1.0985
70	[0 12]	−269.2371	7.5067	0.0000	1	1.000	0.0000
90	[12,11]	−967.3247	15.3563	0.0000	1	0.478	−1.6502
53	[8 18]	−681.2706	12.4209	0.0322	1	0.692	−1.3140
125	[0 12]	−269.1999	7.5067	0.0372	1	1.000	0.0372
28	[23 0]	−1604.0290	22.8215	0.0465	1	0.000	0.0465
34	[18 2]	−1470.8920	21.2585	0.0548	1	0.100	−0.3004
43	[0 11]	−269.1667	7.2106	0.0703	1	1.000	0.0703

**Fig. 6:**
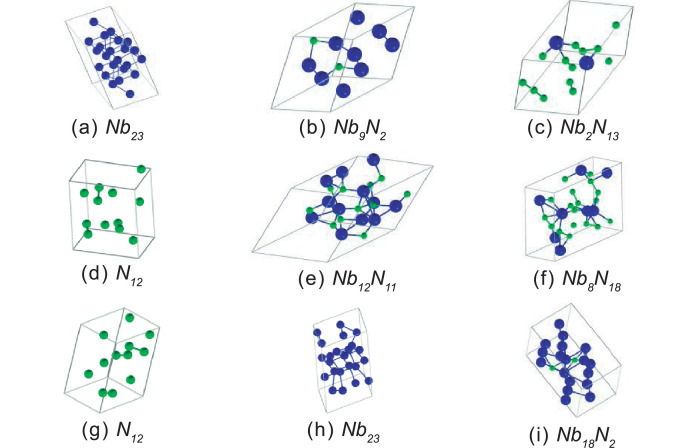
Crystal structures of first nine phases present in the convex hull at 50 GPa. The blue balls are Nb atoms and the green balls are N atoms.

The Fig. 6 shows the crystal structures of the firsts nine phases presented in Table 3.

**Fig. 7:**
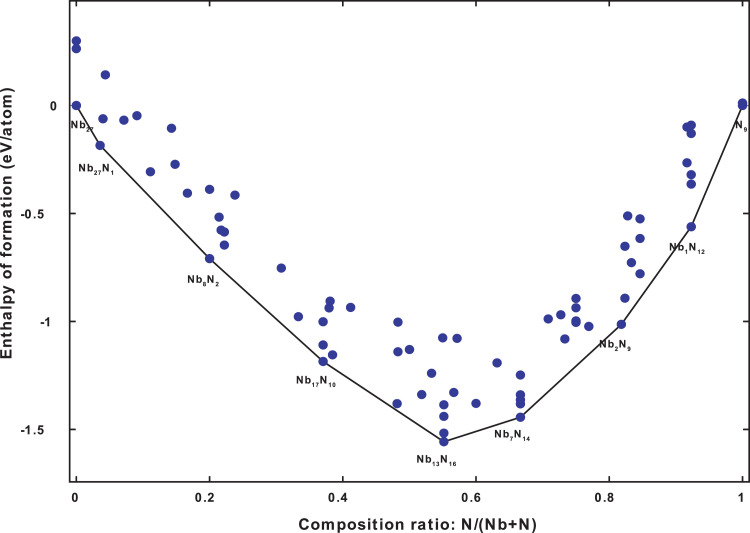
The Convex hull diagram of the Nb-N system at 100 GPa.

For the Case 100 GPa, of the 135 structures generated, 73 of them are present in the convex hull displayed in Fig. 7.

**Fig. 8:**
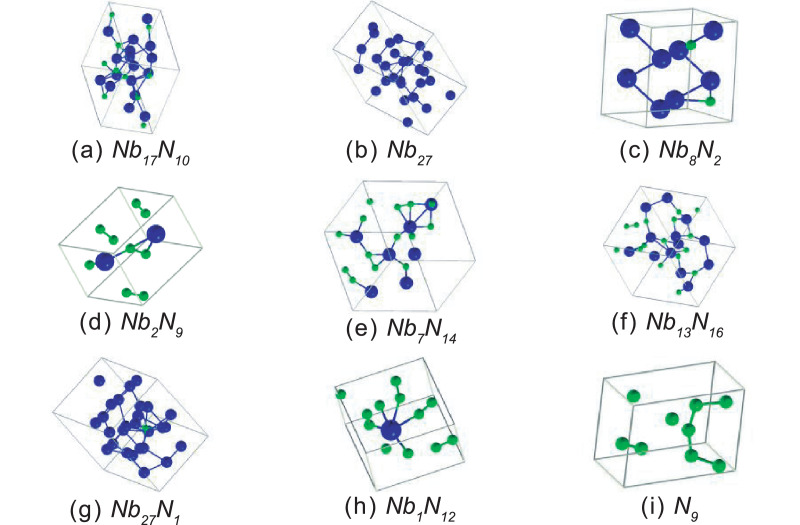
Crystal structures of first nine phases present in the convex hull at 100 GPa. The blue balls are Nb atoms and the green balls are N atoms.

The Table 4 shows the characteristics of the first 10 structures present in the convex hull at 100 GPa.

**Table 4:** Formation enthalpy Y (eV/atom), Composition ratio X, Symmetry S, Fitness F (eV/block), Volumes V(Å^3^/atom), Enthalpies E(eV/atom), and Compositions C for the first 10 structures include in the convex hull of the Nb-N system at 100 GPa.

**ID**	**C**	**E**	**V**	**F**	**S**	**X**	**Y**
26	[17 10]	−1110.9227	17.0387	0.0000	1	0.370	−1.1849
28	[27 0]	−1604.0364	22.8210	0.0000	144	0.000	0.000
46	[8,2]	−1337.8241	19.6973	0.0000	53	0.200	−0.7089
71	[2,9]	−513.0993	13.4886	0.0000	1	0.818	−1.0133
92	[7 14]	−715.7423	17.7711	0.0000	1	0.667	−1.4432
108	[13 16]	−869.2586	14.2094	0.0000	1	0.552	−1.5566
110	[27 1]	−1556.5567	21.1000	0.0000	1	0.036	−0.1848
127	[1,12]	−372.6535	9.3197	0.0000	1	0.923	−0.5611
133	[0 9]	−269.4304	8.0945	0.0000	1	1.000	0.0000
130	[0 11]	−269.4190	8.0942	0.0114	1	1.000	0.0114

The Crystal structures of the firsts nine phases presented in Table 4 are schematically shown in Fig. 2.

For each one of the considered pressures, the phases that make up the corresponding convex hull and the structural representation in a POSCAR file format are contained respectively in the files, extended_convex_hull_ZGPa and extended_convex_hull_POSCARS_ZGPa (with *Z* = 0, 30, 50, and 100), included in the supplementary material.

**Fig. 9:**
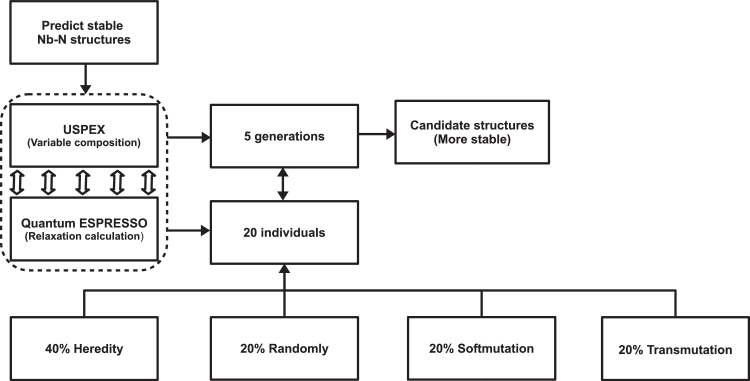
Block diagram to obtain stable and metastable structures of the *Nb*-*N* system.

To obtain the POSCAR files, corresponding to the phases here predicted, the procedure described in the Fig. 9 was followed. In the Fig. 9 it is shown the amount of generations used, the number of individuals or phases created, and the operations implemented to generate the new individuals.

## 2 Experimental design, materials, and methods

Searches for stable and metastable structures of the *Nb*–*N* system were performed using USPEX in its variable composition mode, together with Quantum-ESPRESSO (QE) [11,12], to carry out a relaxation calculation. The prediction calculations were done for five generations with 20 individuals each one. These individuals were produced under the following constraints: 40% produced by inheritance, 20% produced by random, 20% produced by soft mutation and 20% produced by transmutation, each individual can have a maximum of 30 atoms and a minimum of 1. The process for predict stable and metastable structures of the *Nb*-*N* system is described in the following figure.

To perform the process schematically described in the Fig. 9, the input file, INPUT.txt, it must configure and create a folder called Specific. All the parameter to perform the calculation, such as the type of atoms, species, symmetries to explore, pressure, number of individuals (structures that USPEX will predict), number of generations, and any other characteristics that structurally affect the phases that will be generated, are configured in the file INPUT.txt. In addition, in this input file, the computational suite is selected to relax the created structures and the instruction to execute it.

## Declaration of Competing Interest

The authors declare that they have no known competing financial interests or personal relationships, which have, or could be perceived to have, influenced the work reported in this article.

## Appendix Supplementary materials

Supplementary material contains the following: The files INPUT.txt, that contain the settings for each one of the pressures presented in this document, and the folder Specific, in which are stored the QE inputs file identified as qEspresso_options.txt. Besides, a script in BASH Shell labeled as install_QeOcXc, with which the programs QE, Gnuplot, Octave and XCrySDen can be installed, as well as their packages and dependencies. In addition, a script in Python named POSCAR2QE is provided, which converts the USPEX results found in POSCAR format to the QE input file format. In order to recreate or extend the presented results, it is necessary to remove the .txt extension from the QE files and rename the USPEX input files as INPUT.txt Image, application 1
